# An observational study of patients receiving delayed-release budesonide (Nefecon) for immunoglobulin A nephropathy in the United States: A case series of Asian-American patients 

**DOI:** 10.5414/CN112036

**Published:** 2026-08-14

**Authors:** Wei Sun, Terri Madison, Li Yang, Elizabeth Liang, Giancarlo Pesce, Sarah Zaidi, Vishnudas Sarda, Ramin Tolouian, Christopher Ngai

**Affiliations:** 1 Chinatown Kidney Care, New York, NY,; 2 Madison Epi Solutions, LLC, Ann Arbor, MI, USA,; 3 Evidence and Access, Certara, Milan, Italy,; 4 Evidence and Access, Certara, Québec, Canada,; 5 Evidence and Access, Certara, Hyderabad, India, and; 6 Calliditas Therapeutics, New York, NY, USA

**Keywords:** delayed-release budesonide, IgA nephropathy, real-world study, renal outcomes, Nefecon

## Abstract

Introduction: IgA nephropathy (IgAN) is a leading cause of glomerulonephritis worldwide with variability by race and ethnicity. This study aimed to provide insights on the treatment journey and clinical outcomes in Asian-American patients with IgAN treated with delayed-release budesonide (Nefecon) in a real-world setting. Materials and methods: In this observational cohort study, a total of 45 consecutive patients with IgAN treated with Nefecon at Chinatown Kidney Care (CKC) (New York, NY, USA) were included. Study outcomes included treatment patterns and clinical outcomes (time to kidney failure, estimated glomerular filtration rate (eGFR), and urine protein-to-creatinine ratio (UPCR)). All variables were summarized descriptively. Subgroup analyses were performed among patients who were treated for ≥ 9 months vs. those treated for < 9 months. Results: Overall, after 9 months of treatment, eGFR remained stable and UPCR decreased by an average of 19%. Improved clinical outcomes were reported among those who were treated for ≥ 9 months (preservation of eGFR and 26% reduction in UPCR) compared with those treated for < 9 months (decline in eGFR and ~ 3% reduction in UPCR). Nefecon was well tolerated with no severe or unexpected adverse events reported. Conclusion: Real-world IgA nephropathy treatment-associated outcomes with Nefecon in Asian-American patients for proteinuria and kidney function were consistent with those observed in the NefIgArd clinical trial. The reduction in proteinuria and improvement in kidney function was greater in patients who completed the 9-month course of therapy. These findings reveal an opportunity for further education among nephrologists regarding the recommended duration of treatment with Nefecon.

## Introduction 

Immunoglobulin A nephropathy (IgAN) is an immune-mediated glomerular disease and a leading cause of chronic kidney disease and failure [1, 2, 3, 4, 5], with a particularly high incidence among Asian and Asian-American populations [6, 7]. Despite optimized supportive care, many patients remain at risk of progressive loss of kidney function, underscoring the need for disease modifying therapies [2, 8]. 

The 2021 Kidney Disease Improving Global Outcomes (KDIGO) clinical guidelines recommended optimized supportive care as the mainstay of therapy in IgAN, including lifestyle modifications, blood pressure control, and renin-angiotensin system blockade to reduce proteinuria [9]. After conditional approval in December 2021, in December 2023, the U.S. Food and Drug Administration (FDA) granted full approval for a targeted delayed-release capsule formulation of budesonide for adults with primary IgAN at risk of disease progression [5], based on the 2-year results of the randomized, placebo-controlled phase-3 NefIgArd study, which demonstrated a sustained reduction in proteinuria and improvement in glomerular filtration rate (eGFR) [10, 11]. The formulation is marketed in the U.S. as Tarpeyo, Calliditas Therapeutics AB, Stockholm, Sweden, and is referred to as Nefecon throughout this manuscript. Reflecting these findings, the KDIGO 2025 guidelines recommend a 9-month course of Nefecon in accordance with the current U.S. Prescribing Information, providing a clinically relevant benchmark for treatment duration [12, 13]. 

Evidence on the use of Nefecon in routine practice, particularly among Asian-American patients in the United States, remains limited. This study describes the demographic and clinical characteristics, treatment patterns including real-world duration of therapy, and kidney outcomes of Asian-American patients with IgAN treated with Nefecon at a single nephrology practice in the United States. 

## Materials and methods 

### Study design 

This observational study included an exposure-based case series of consecutive patients with IgAN treated with Nefecon at the CKC practice. The observation period for each patient extended from the time of IgAN diagnosis to up to 2 years following the initiation of Nefecon based on data availability. Patients were followed for a maximum of 2 years, unless follow-up ended earlier due to death, loss to follow-up, or the end of available data, with data abstraction cutoff no later than November 30, 2023 (Supplemental Figure S1). Data were collected retrospectively from patients’ electronic medical records (EMR) using an electronic case report form (eCRF). If a patient was diagnosed before being seen at the CKC, data were collected using the first date available in the CKC EMR for demographic or disease characteristics and at Nefecon initiation for other clinical characteristics. 

Data for this study were collected as a limited de-identified dataset. The study protocol was submitted along with the eCRF to a central Institutional Review Board and was exempted under U.S. Health and Human Services code 45 CFR 46.104(d)(4). 

### Patient population 

Eligible patients included those who received at least 1 dose of Nefecon at the CKC. Patients must have received at least 1 dose of Nefecon at the CKC before March 1, 2023, to allow patients to have received treatment for at least one 9-month course as of the data abstraction cutoff date. Patients were excluded if they had kidney failure (defined as end-stage kidney disease, were on chronic dialysis, or had received kidney transplant) at or before initiation of Nefecon treatment. 

### Study outcomes 

The study aimed to describe patient demographics, disease, and clinical characteristics; IgAN-directed treatment journey, that is, treatment pattern of Nefecon and use of other IgAN-directed treatments prior/during/after initiating treatment with Nefecon; clinical outcomes of disease progression such as time to kidney failure/survival and other biomarkers of progression (eGFR and urine protein-to-creatinine ratio (UPCR)); and adverse events (AEs) that occurred during treatment with Nefecon. 

Treatment duration was as documented in routine clinical practice, and patients received real-world treatments including Nefecon at doses and durations based on clinical judgment of the treating physician. 

### Statistical analysis 

All variables were summarized descriptively. Categorical variables were summarized using frequency counts and percentages (%), while continuous variables were summarized using mean and standard deviation (SD). No replacement or imputation methods were performed for any missing values. The primary analysis population included all patients who met the study eligibility criteria. Subgroup analyses were performed among patients who received the recommended duration of therapy per the U.S. Prescribing Information (i.e., at least one 9-month course of Nefecon) compared with patients who received treatment for less than 9 months [12]. All analyses were performed using SAS Version 9.4. 

## Results 

As of March 1, 2023, 45 patients who met eligibility criteria were included in this study (Figure 1). 

### Patient characteristics 

Overall, the mean age at initiation of Nefecon was 48.1 years (SD = 13.6); 58% of patients were male, and all except 1 patient with unknown/undisclosed race were Asian-American. A total of 59% of patients had chronic kidney disease (CKD) stage ≥ 3 (Table 1). Patients who received ≥ 9 months of treatment (n = 30) were younger, and most had better renal function at initiation of treatment compared with patients who did not receive a full 9-month course (n = 15) (Table 1). 

### Treatment patterns of patients with IgAN at CKC 

Among all patients, the average time from IgAN diagnosis to initiation of Nefecon was 5.6 years (SD = 6.5). Overall, patients received Nefecon for an average of 9.3 months (SD = 4.2). The average time among those who received treatment for ≥ 9 months was 11.7 months (SD = 2.7). A total of 67% of patients received Nefecon for ≥ 9 months, while 33% discontinued prior to completing the full 9-month course (Table 2). The reasons for early treatment discontinuation were adverse events (n = 5), lack of efficacy (n = 3), or disease progression (n = 1), while 3 patients discontinued Nefecon early after their UPCR normalized. 

At the time of treatment initiation, 87% of patients were receiving an angiotensin receptor blocker (ARB), and 53, 29, and 22% of patients were receiving mycophenolate mofetil, systemic corticosteroids, and sodium-glucose co-transporter-2 inhibitors, respectively. Overall, 18% of patients also received sparsentan after starting Nefecon. Similar treatment patterns were observed for the 2 subgroups, except that 97% of patients treated for ≥ 9 months were receiving an ARB at the time of treatment initiation, and 40% of patients treated for < 9 months received sparsentan after starting Nefecon (Figure 2). 

### Clinical outcomes of disease progression 

Overall, mean eGFR at the time of treatment initiation of Nefecon was 61.9 mL/min/1.73m^2^ (SD = 31.2); among patients treated for ≥ 9 months and < 9 months, the mean eGFR was 68.4 and 49.1 mL/min/1.73m^2^, respectively. After 9 months of treatment, eGFR remained stable (mean change (SD): 0.9 (10.3) mL/min/1.73m^2^) among the study population; patients who were treated for ≥ 9 months had an increase of 3.6 mL/min/1.73m^2^ (SD = 10.6), while those who were treated for < 9 months had a decrease of 3.5 mL/min/1.73m^2^ (SD = 8.3) (Figure 3). 

On average, patients were followed up for 14.9 months (SD = 3.3). One patient had kidney failure during follow-up. A total of 13% of patients had a 30% reduction in eGFR (7% among those treated for ≥ 9 months and 27% among those treated for < 9 months). Additionally, 7% of patients had a 40% eGFR reduction (3% (treated ≥ 9 months) and 13% (treated < 9 months)), and 4% had a 50% reduction (3% (treated ≥ 9 months) and 7% (treated < 9 months)) (Table 3). 

The average UPCR was 1.48 mg/mg at the time of treatment initiation; among patients treated for ≥ 9 months and < 9 months, the mean UPCR was 1.56 and 1.29 mg/mg, respectively. At 9 months, the overall UPCR decreased by an average of 19%; patients who were treated for ≥ 9 months showed a 26% reduction in UPCR, while those who were treated for < 9 months had a 3.3% reduction (Figure 4). 

### Safety 

Overall, 7 patients (16%) reported AEs, all of which were mild (n = 6) or moderate (n = 1) (Table 4). In 5 cases, AEs led to permanent discontinuation of Nefecon (edema/swelling in 4 patients and blood pressure increase in 1 patient). All AEs were fully resolved at the time of data abstraction cutoff. 

## Discussion 

This longitudinal case series study describes the real-world use of Nefecon in patients of Chinese descent with IgAN and shows that treatment-associated changes in proteinuria and kidney function were directionally consistent with those observed in the pivotal phase-3 NefIgArd trial [11]. Patients who discontinued Nefecon before completing 9 months of treatment showed smaller improvements in proteinuria and eGFR. 

The more favorable kidney function and proteinuria trajectories observed in patients treated for ≥ 9 months compared with those treated for <9 months should be interpreted cautiously because these exploratory subgroup comparisons are based on realized treatment duration and thus reflect differential treatment persistence rather than randomized exposure: patients who completed a full 9-month course were generally younger, had better baseline eGFR, and were more likely to tolerate or appear to respond to therapy, whereas those with a more severe or unstable course often discontinued earlier. Accordingly, these descriptive differences cannot be taken as evidence that longer treatment duration per se causes better outcomes. 

Although these differences are likely influenced by baseline clinical characteristics and physician-driven treatment decisions, they are consistent with the 9-month treatment course evaluated in clinical trials and recommended in the U.S. Prescribing Information and recent guidelines [10, 11, 12, 13]. 

The clinically relevant reduction in proteinuria with preserved eGFR observed in this study, particularly among patients treated for ≥ 9 months, is consistent with the findings from the China cohort of the NefIgArd trial [14]. These results reinforce the efficacy profile of Nefecon demonstrated in clinical trials [10, 11, 14, 15]. In this study, the most notable reduction in proteinuria was observed after ~ 6 months of treatment, which is consistent with observations from the global and China-based cohorts of the NefIgArd trial [10, 11, 14]. These findings suggest that the full therapeutic benefit of Nefecon may not be observed until patients have completed at least 6 to 9 months of therapy, and that discontinuing treatment based on early assessments of proteinuria may represent a missed opportunity for sustained proteinuria reduction and longer-term renal benefit. 

In this study, Nefecon was generally well tolerated, with no severe or unexpected adverse events, and all events resolving without complications. Discontinuations due to AEs occurred in 5 patients (11%), a proportion comparable to that reported in the NefIgArd study (9%), supporting the favorable safety profile observed in clinical trials and its applicability in routine practice [10, 11, 14]. It has been known that traditional systemic corticosteroids have been associated with higher rates of serious AEs, including severe infections, excess hospitalizations, impaired glucose tolerance, and death [16, 17, 18]. Nefecon is formulated to deliver the drug directly to the gut-associated lymphoid tissue in the terminal ileum where a high concentration of Peyer’s patches are located, with less than 10% systemic exposure [19, 20, 21, 22]. The higher local glucocorticoid potency and low systemic bioavailability of Nefecon may contribute to a more favorable safety profile compared with prednisone. 

In this cohort, the mean age at Nefecon initiation was 48 years, which is somewhat higher than the median age at IgAN diagnosis reported in large biopsy-based series but broadly consistent with real-world treatment cohorts in which disease-modifying therapies, such as SGLT2 inhibitors, are typically introduced in the fourth to fifth decade of life [23, 24]. This pattern likely reflects routine practice in a specialty setting, where Nefecon is often initiated several years after diagnosis, once patients have demonstrated persistent proteinuria and are perceived to be at higher risk of progression. 

Because product labels and KDIGO guidance at the time these patients received Nefecon (pre-2024) did not support routine concomitant use of Nefecon and sparsentan, patients who initiated sparsentan at this center generally discontinued Nefecon. This treatment sequencing likely underlies the higher proportion of shorter Nefecon duration among patients who received sparsentan and should be interpreted as reflecting confounding by indication and physician-directed switching rather than a causal effect of sparsentan on Nefecon persistence. 

To our knowledge, this is one of the first real-world studies that focused on an Asian-American population with IgAN in the U.S. One of the key strengths of this study is the biopsy-proven diagnosis of IgAN. Additionally, the focus on a predominantly Asian-American population, an ethnic group with a disproportionately high incidence of IgAN, provides important insights into disease progression and management in a historically underrepresented group. Furthermore, the consistency of the standard of care provided at the CKC enhances the reliability of the findings. 

This study has several inherent limitations typical of real-world case-series research. It is a relatively small, single-center observational study without a comparator group, and the population exhibited substantial heterogeneity in background IgAN management, including widespread use of systemic corticosteroids, mycophenolate mofetil, ARBs, and SGLT2 inhibitors. While such heterogeneity reflects contemporary real-world practice, it complicates attribution of outcomes to any specific intervention. Moreover, prior or concomitant use of systemic corticosteroids and other IgAN-directed therapies represents a potential source of confounding, as some of the observed early treatment-associated changes in proteinuria and kidney function may partially reflect delayed effects of these background therapies rather than Nefecon alone. 

The mean follow up of approximately 15 months is sufficient to characterize intermediate-term treatment-associated changes in proteinuria, eGFR, and early safety events – similar to the time frame in which clinical trials of Nefecon have shown significant effects [10, 11, 14] – but remains too short to define long-term renal outcomes such as kidney failure in IgAN. 

Despite these limitations, the study provides meaningful real-world insights into the use of Nefecon in a high-risk, historically underrepresented population. 

## Conclusion 

Overall, the findings from this real-world case series are consistent with the results of the NefIgArd clinical trial and provide supportive evidence on the recommended duration of use and tolerability of Nefecon in patients with IgAN, including high-risk populations such as Asian-Americans. In this cohort, treatment with Nefecon was associated with favorable trends in proteinuria and kidney function over time, especially for patients treated for the recommended 9-month duration, consistent with outcomes observed in randomized clinical trials. Although the descriptive, non-comparative design of this study precludes causal inference, these results contribute meaningful real-world insights into the effectiveness and tolerability of Nefecon in routine clinical practice. 

## Acknowledgments 

Medical writing support was provided by Preetinder Kaur of Certara. 

## Authors' contributions

Concept and design: TM, GP, SZ, RT, and CN. 

Acquisition, analysis, or interpretation of data: WS, TM, LY, EL, GP, SZ, VS, RT, and CN. 

Statistical analysis: GP and VS. 

Each author contributed important intellectual content during manuscript drafting or revision and accepts accountability for the overall work by ensuring that questions pertaining to the accuracy or integrity of any portion of the work are appropriately investigated and resolved. All authors agreed to the final submitted version. 

## Funding 

The study was funded by Calliditas Therapeutics (now part of Asahi Kasei Corporation). 

## Conflict of interest 

WS, LY, and EL received funding from Calliditas Therapeutics for the conduct of this study. TM reports receiving funding from Calliditas Therapeutics for the design, conduct, analysis, and reporting of this study when she was an employee of Certara, and subsequently through her company Madison Epi Solutions LLC. GP, SZ, and VS report that their institution (Certara) received funding from Calliditas Therapeutics for the study design, conduct, analysis, reporting, and medical writing support. RT and CN were employees of Calliditas Therapeutics, now part of Asahi Kasei Corporation, during the conduct of the study. 

**Figure 1:**
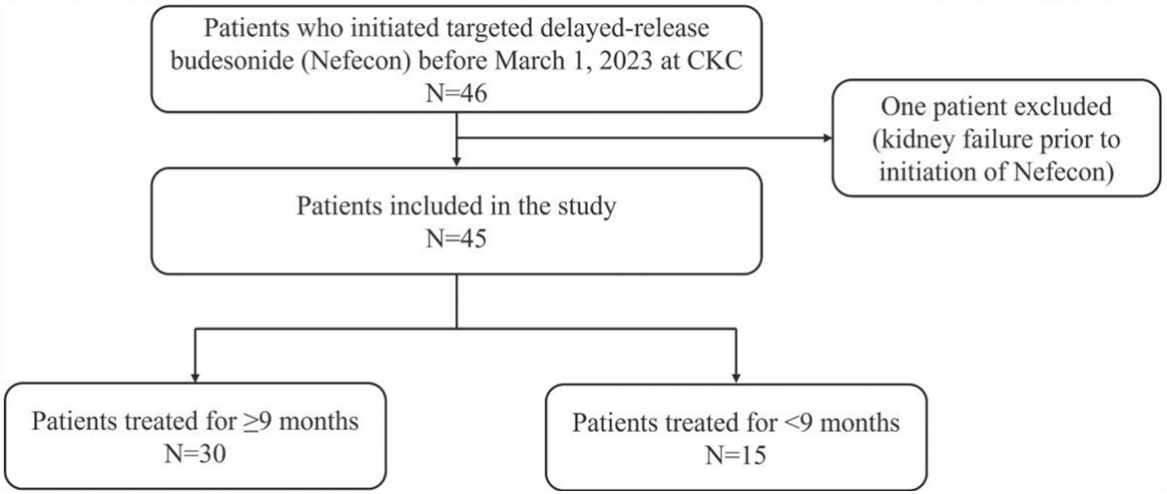
Patient flow diagram. CKC = Chinatown Kidney Care practice (New York, NY, USA).

**Table 1.** Patient characteristics.

	All patients (N = 45)	Received Nefecon for ≥ 9 months (N = 30)	Did not receive Nefecon for 9 months (N = 15)
Age at IgAN diagnosis, years, mean (SD)	42.5 (13.0)	40.6 (12.4)	46.3 (13.6)
Age at treatment initiation with Nefecon, years, mean (SD)	48.1 (13.6)	46.5 (12.7)	51.5 (15.2)
Sex, male, n (%)	26 (57.8)	17 (56.7)	9 (60.0)
Race^†^, Asian, n (%)	44 (97.8)	30 (100.0)	14 (93.3)
Current smokers, n (%)	29 (64.4)	19 (63.3)	10 (66.7)
BMI, kg/m^2^, mean (SD)	24.8 (4.1)	25.0 (3.9)	24.4 (4.6)
LDL cholesterol, mg/dL, mean (SD)	124.5 (45.7)	121.5 (33.0)	130.4 (64.6)
HDL cholesterol, mg/dL, mean (SD)	59.4 (16.7)	58.1 (16.3)	61.9 (17.8)
Hypertension, n (%)	36 (80.0)	23 (76.7)	13 (86.7)
Diabetes, n (%)	5 (11.1)	3 (10.0)	2 (13.3)
CKD stage ≥ 3, n (%)	26 (59.0)*	14 (48.0)*	12 (80.0)

*One patient had missing information on CKD stage. †One patient had “Other or Undisclosed” race. BMI = body mass index; CKD = chronic kidney disease; HDL = high-density lipoprotein; IgAN = immunoglobulin A nephropathy; LDL = low-density lipoprotein; SD = standard deviation.

**Table 2.** Use of Nefecon in patients with IgAN at the CKC.

	All patients (N = 45)	Received Nefecon for ≥ 9 months (N = 30)	Did not receive Nefecon for 9 months (N = 15)
Diagnosis to Nefecon initiation, years, mean (SD)	5.6 (6.5)	5.9 (7.4)	5.1 (4.4)
Nefecon dose, mg/day, mean (SD)	16.0 (0.0)	16.0 (0.0)	16.0 (0.0)
Nefecon treatment duration, months, mean (SD)	9.3 (4.2)	11.7 (2.7)	4.6 (2.3)
Received 2 cycles*of Nefecon, n (%)	1 (2.3)	1 (3.4)	0 (0.0)
Reason for discontinuing Nefecon, n (%)			
Adverse event	–	–	5 (33.3)
Disease progression	–	–	1 (6.7)
Lack of effect	–	–	3 (20.0)
Other^#^	–	–	5 (33.3)
Unknown	–	–	1 (6.7)

*One cycle of Nefecon corresponds to a 9-month course. ^#^Of these 5 patients, 3 patients discontinued as their UPCR went back to normal, 1 patient discontinued due to dose administration (difficult to swallow), and 1 patient provided no reason. CKC = Chinatown Kidney Care practice (New York, NY) in the United States; IgAN = immunoglobulin A nephropathy; SD = standard deviation; UPCR = urine protein-to-creatinine ratio.

**Figure 2:**
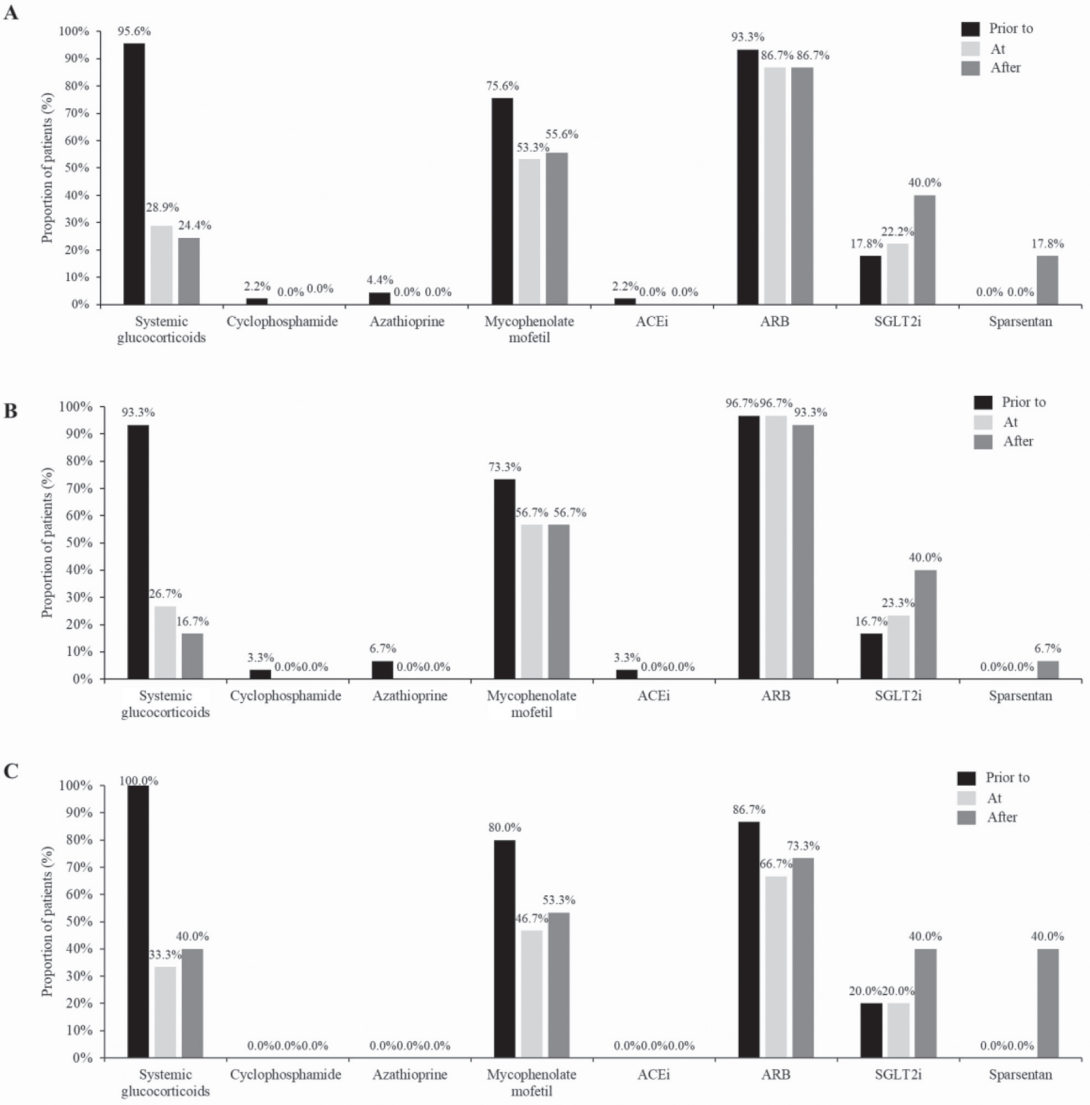
Medications received prior to, at, and after initiating treatment with Nefecon in patients with IgAN at the CKC. A: All patients (N = 45). B: Patients with ≥ 9 months of treatment (N = 30). C: Patients with < 9 months of treatment (N = 15). ACEi = angiotensin-converting enzyme inhibitor; ARB = angiotensin receptor blocker; CKC = Chinatown Kidney Care practice (New York, NY) in the United States; IgAN = immunoglobulin A nephropathy; SGLT2i = sodium-glucose co transporter-2 inhibitor.

**Figure 3:**
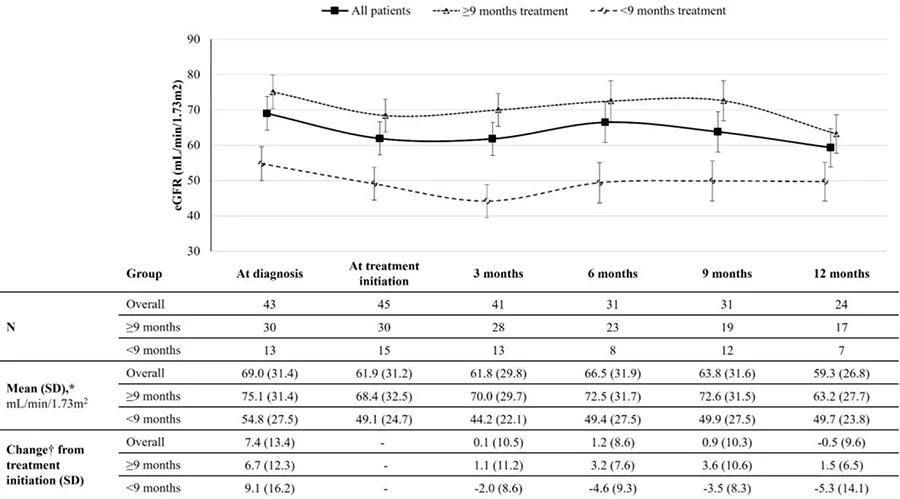
Estimated glomerular filtration rate distribution at diagnosis, at treatment initiation, and after treatment initiation with Nefecon. *Among all patients. †Among patients with measurement at both time points. eGFR = estimated glomerular filtration rate; SD = standard deviation.

**Table 3.** Clinical outcomes in patients with IgAN treated with Nefecon at the CKC.

	All patients (N = 45)	Received Nefecon for ≥ 9 months (N = 30)	Did not receive Nefecon for 9 months (N = 15)
Follow-up time*, months, mean (SD)	14.9 (3.3)	14.4 (3.3)	15.8 (3.2)
Total follow-up, person-months	669	432	237
Kidney failure, n (%)	1 (2.2)	0	1 (6.7)
30% eGFR reduction, n (%)	6 (13.3)	2 (6.7)	4 (26.7)
40% eGFR reduction, n (%)	3 (6.7)	1 (3.3)	2 (13.3)
50% eGFR reduction, n (%)	2 (4.4)	1 (3.3)	1 (6.7)

*From treatment initiation with Nefecon to last data available. CKC = Chinatown Kidney Care practice (New York, NY) in the United States; eGFR = estimated glomerular filtration rate; IgAN = immunoglobulin A nephropathy; SD = standard deviation.

**Figure 4:**
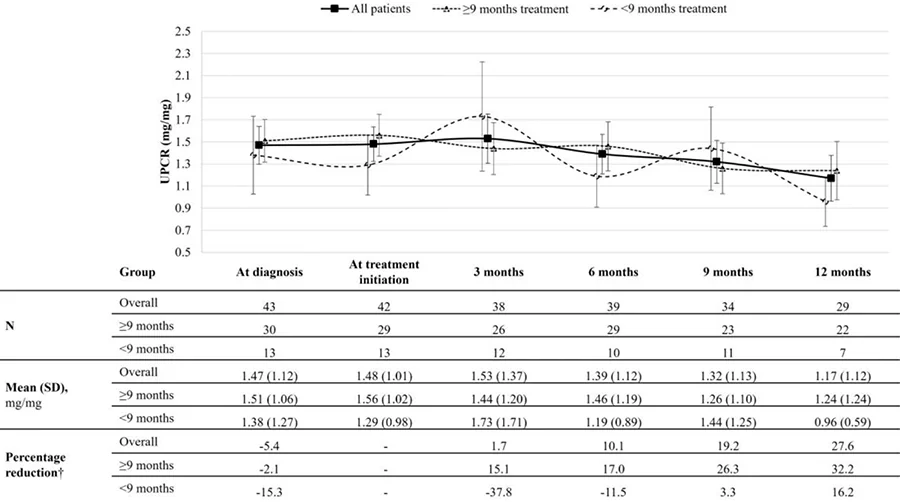
Urine protein-to-creatinine ratio distribution at diagnosis, at treatment initiation, and after treatment initiation with Nefecon. †Compared with treatment initiation, among patients with measurement at both timepoints. SD = standard deviation; UPCR = urine protein-to-creatinine ratio.

**Table 4.** Adverse events.

	All patients (N = 45)	Received Nefecon for ≥ 9 months (N = 30)	Did not receive Nefecon for 9 months (N = 15)
Any AE, n (%)	7 (15.6)	2 (6.7)	5 (33.3)
AE severity, n (%)			
Mild	6 (85.7)	2 (100.0)	4 (80.0)
Moderate	1 (14.3)	0	1 (20.0)
Severe	0	0	0
AE outcome, n (%)			
Ongoing	0	0	0
Resolved	7 (100.0)	2 (100.0)	5 (100.0)
Resolved with sequelae	0	0	0
Action taken regarding Nefecon, n (%)			
None	1 (14.3)	1 (50.0)	0
Dose modification	1 (14.3)	1 (50.0)	0
Permanently discontinued	5 (71.4)	0	5 (100.0)

AE = adverse event.

## Supplemantal meterial

Supplemental material Supplemental figure
