# Supplementary material for: An observational study of patients receiving delayed-release budesonide (Nefecon) for immunoglobulin A nephropathy in the United States: A case series of Asian-American patients
Source: Clin Nephrol. 2026 Aug 14;106(4):276–85. doi: 10.5414/CN112036 (PMC13621273; doi:10.5414/CN112036)
Supplement: Supplementary file 1 [file clinnephrol-106-276-S01.pdf]

Figure S1. Study design

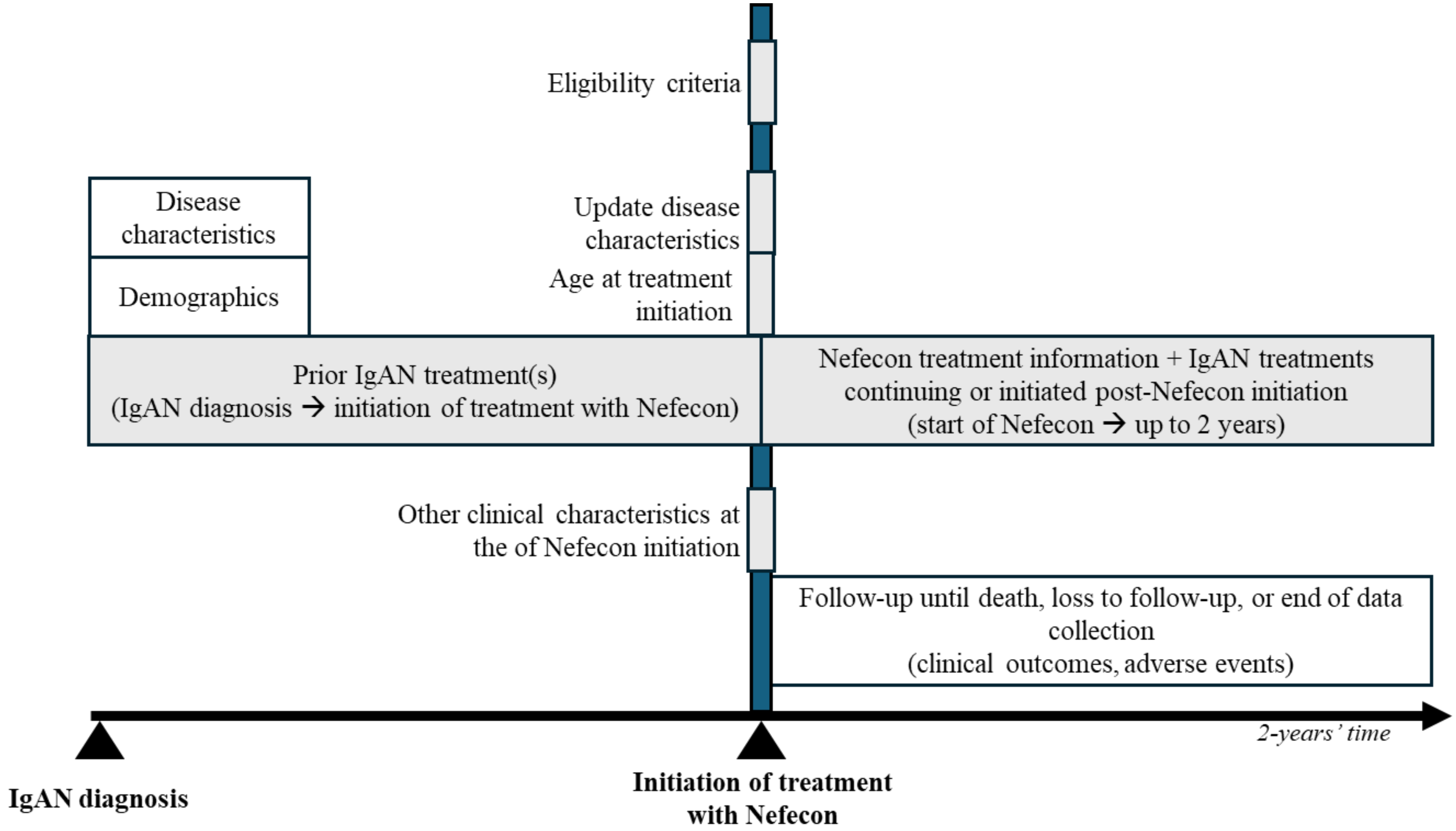

Abbreviations: IgAN, immunoglobulin A nephropathy.
